# Supplementary material for: Model‐Driven Engineering of Yarrowia lipolytica for Improved Microbial Oil Production
Source: Microb Biotechnol. 2025 Mar 20;18(3):e70089. doi: 10.1111/1751-7915.70089 (PMC11925697; doi:10.1111/1751-7915.70089)
Supplement: Supplementary file 1 — Data S1. [file MBT2-18-e70089-s001.docx]

**Supplementary Material**

**
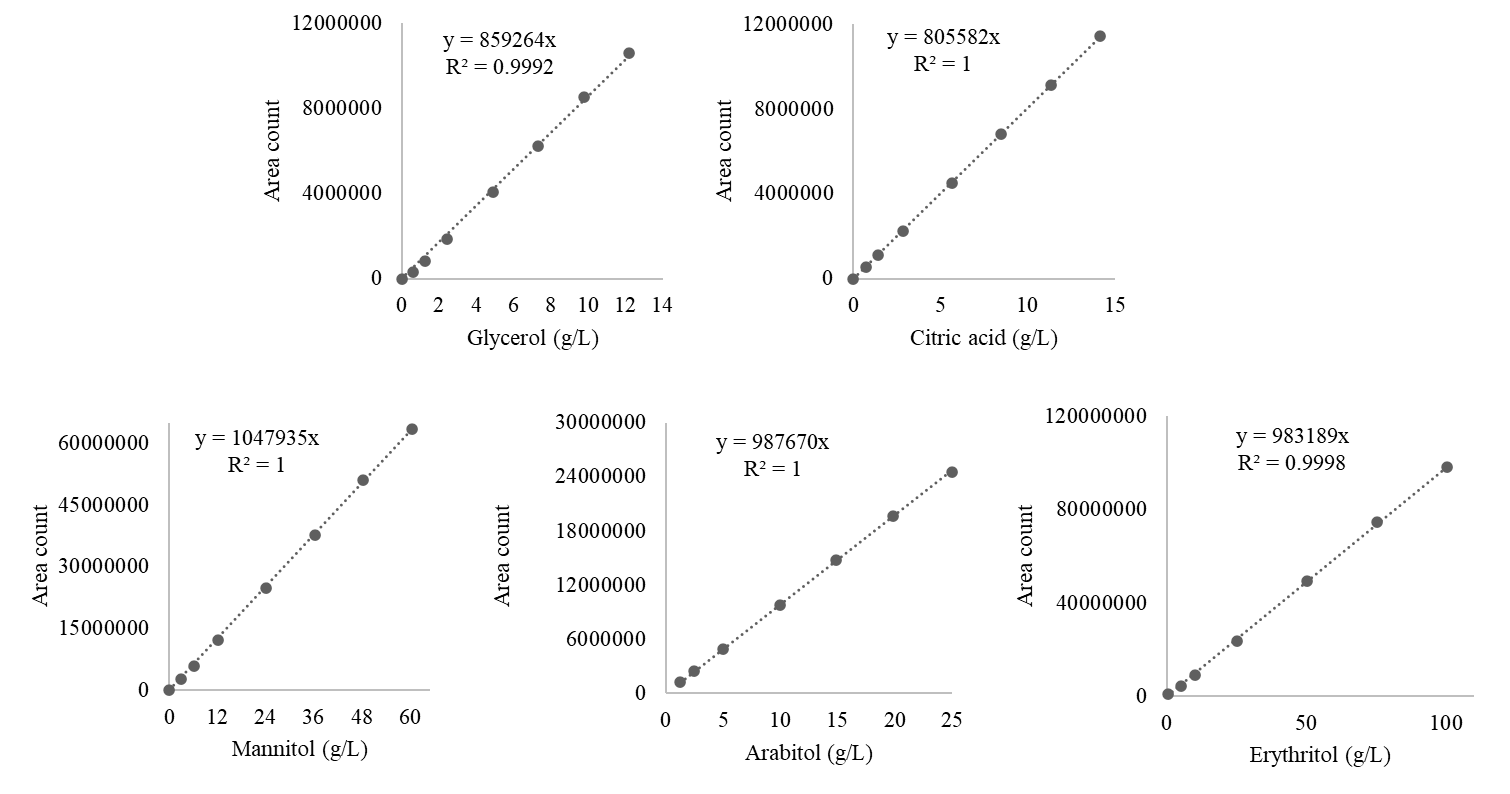
**

**Figure S1.** Calibration curve of glycerol, citric acid, mannitol, arabitol, and erthritol for calculating their concentrations in the medium.

**
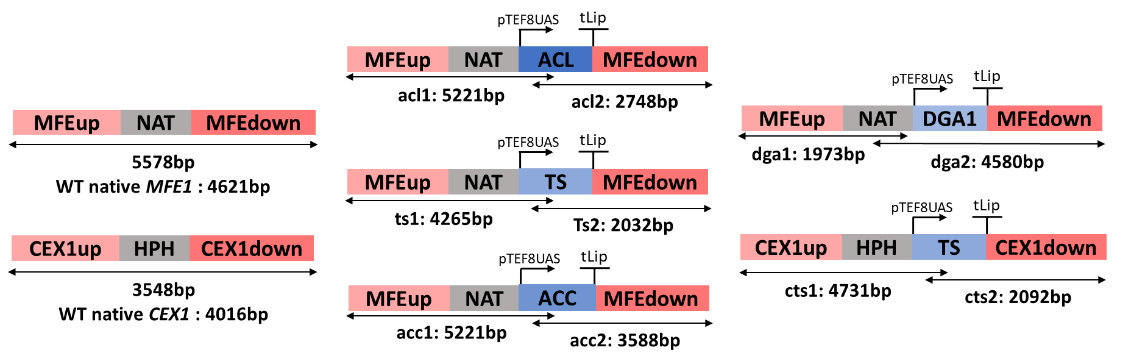
**

**
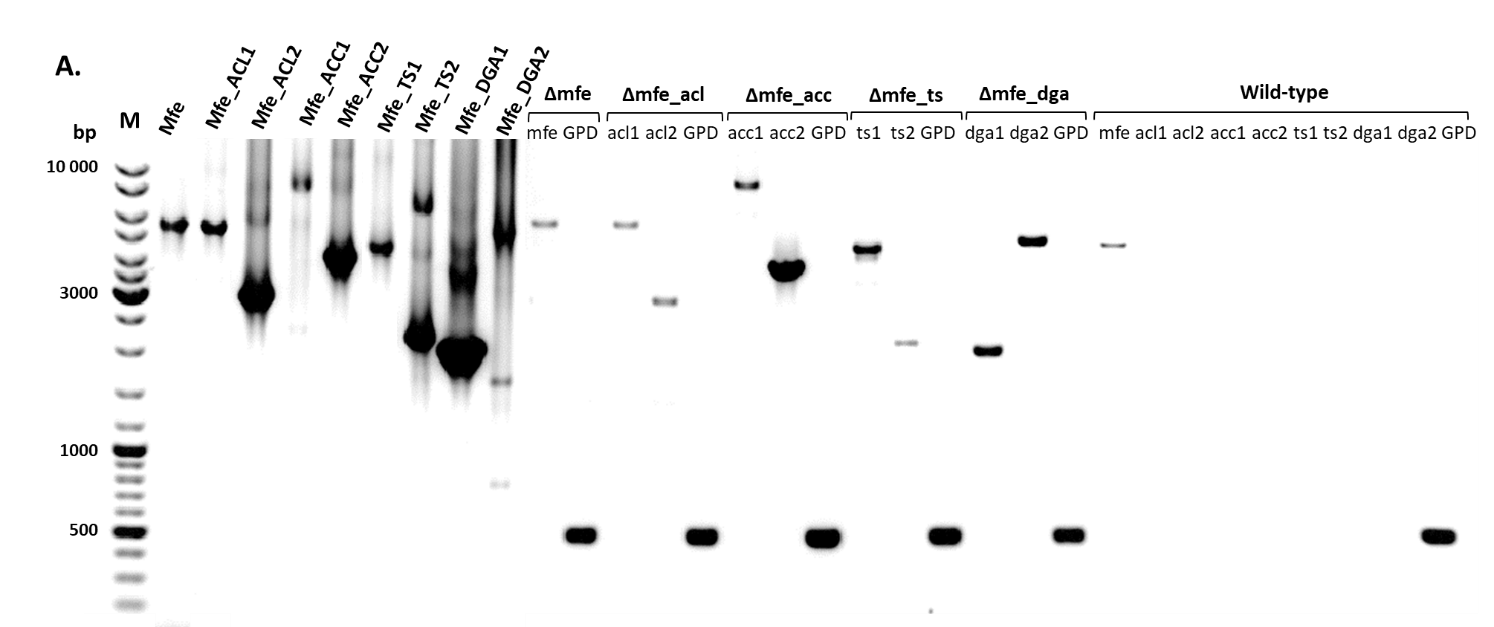
**

**
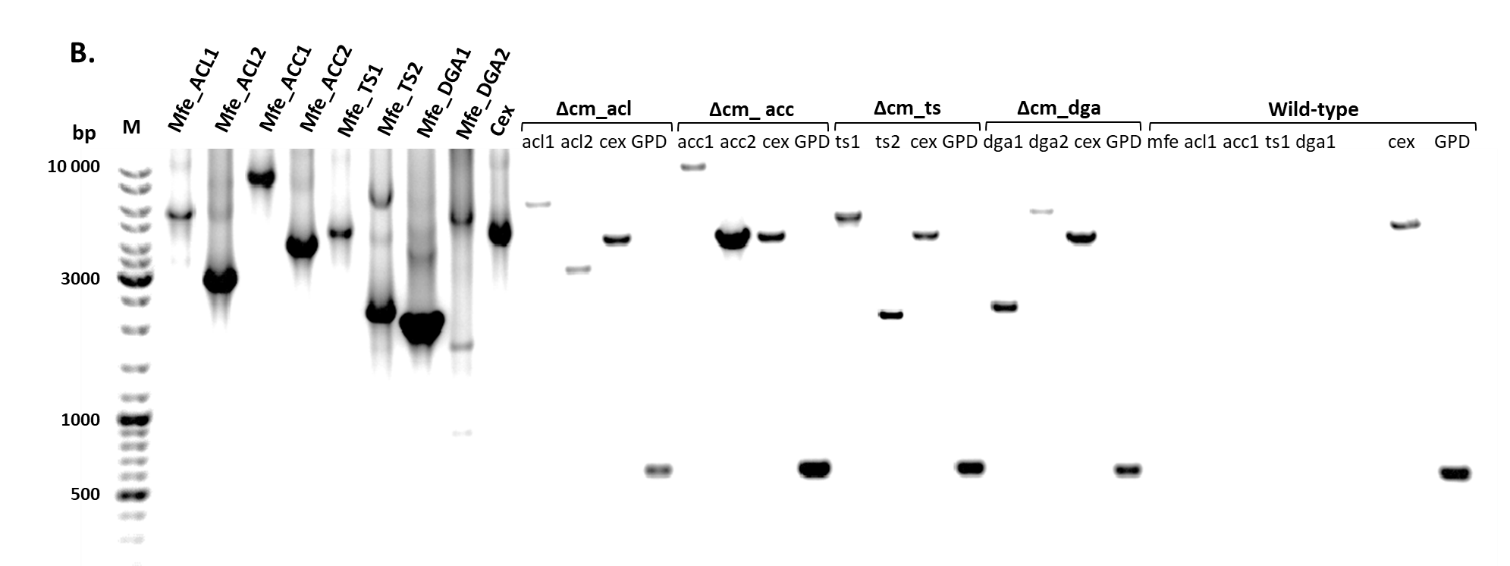
**

**
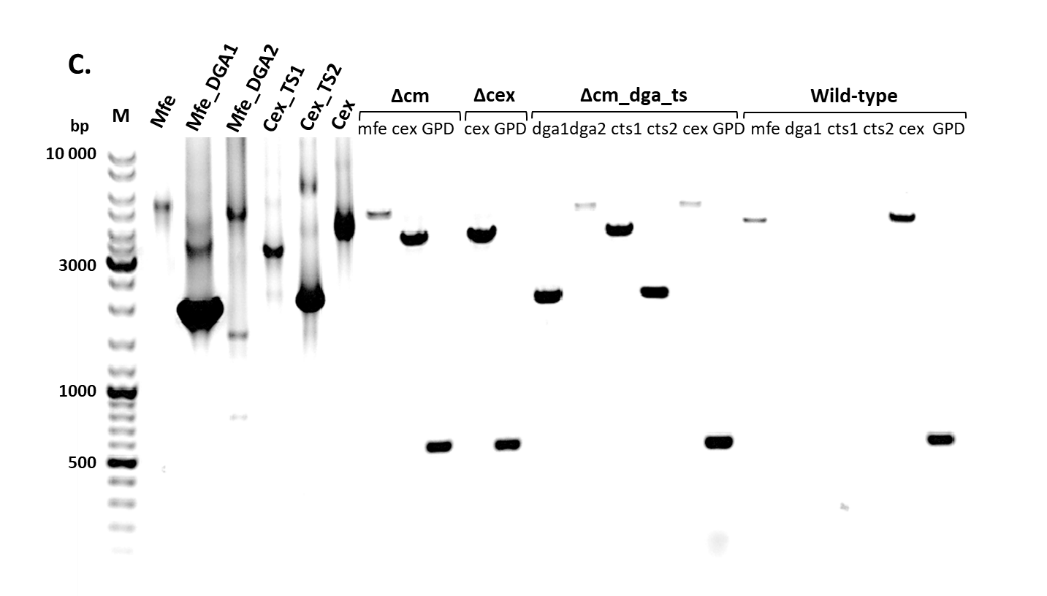
**

**Figure S2. Colony PCR products were run on 1 % agarose gel.** Positive controls were prepared by using corresponding primers and plasmids. Quality of gDNA in PCR reaction was assessed by using primers specific for the glyceraldehyde-3-phosphate dehydrogenase gene (*GPD,* Accession: XM_500444). Expected lengths were indicated per construct. **A)** Agarose gel photo of *Δmfe* strains **B)** Agarose gel photo of *Δcex_Δmfe* strains **C)** Agarose gel photo of *Δcex_Δmfe_dga_ts*.

**Table S1.** Primers designed for cPCR and PCR amplification of genes.

| **Primer Name** | **Sequence** | **Usage** |
| --- | --- | --- |
| ACL_Bsa_D_Fw | gggGGTCTCtAATGATGGCTACCAAGGCTATACGCG | Forward primer for the amplification of *ACL*, inserting the BsaI restriction site. |
| ACL_Bsa_E_Rv | cccGGTCTCtTAGATAAGGGCCAGCGAGGACGCG | Reverse primer for the amplification of *ACL*, inserting the BsaI restriction site. |
| ACC_Bsa_D_Fw | gggGGTCTCtAATGATGGCCGCTGTCGCTCCCAGTG | Forward primer for the amplification of *ACC*, inserting the BsaI restriction site. |
| ACC_Bsa_E_Rv | cccGGTCTCtTAGATTACTGCTGCGAGAGCGAGGCGAT | Reverse primer for the amplification of *ACC*, inserting the BsaI restriction site. |
| TS_Bsa_D_Fw | GGGGGTCTCTAATGATGGCCCAGACGCGCTACTTCTC | Forward primer for the amplification of *TS*, inserting the BsaI restriction site. |
| TS_Bsa_E_Rv | cccGGTCTCtTAGACTACGCCGGGGTGGGCTTCTCG | Reverse primer for the amplification of *TS*, inserting the BsaI restriction site. |
| DGA_D_BsaI_Fw | gggGGTCTCtAATGACTATCGACTCACAATACTAC | Forward primer for the amplification of *DGA*, inserting the BsaI restriction site. |
| DGA_E_BsaI_Rv | cccGGTCTCtTAGATTACTCAATCATTCGGAACTCTGGGGCTC | Reverse primer for the amplification of *DGA*, inserting the BsaI restriction site. |
| Cex1Up_Bsa_A_Fw | gggGGTCTCtGCCTGCGGCCGCTAAACTGGCGCTTACAAATGGCTG | Forward primer for the amplification of homologous *CEX1* up, inserting the BsaI restriction site. |
| Cex1Up_Bsa_B_Rv | cccGGTCTCtACCTGACCCAGGCGGCAGTCCAGAAACC | Reverse primer for the amplification of homologous *CEX1* up, inserting the BsaI restriction site. |
| Cex1Down_Bsa_C_Fw | gggGGTCTCtACGGGCGGCCGCACACTGCTGTCAACACTCCTGC | Forward primer for the amplification of homologous *CEX1* down, inserting the BsaI restriction site. |
| Cex1Down_Bsa_L_Fw | gggGGTCTCtGAGTACACTGCTGTCAACACTCCTGC | Forward primer for the amplification of homologous *CEX1* down, inserting the BsaI restriction site. |
| Cex1Down_Bsa_M_Rv | cccGGTCTCtCGCAGCGGCCGCAGACCGGCCGCCAACAGCAAAGAT | Reverse primer for the amplification of homologous *CEX1* down, inserting the BsaI restriction site. |
| Mfe_Up_Fw | ATGTTGACTCCAGCAGCGAA | Forward primer to check knock-out of *MFE* gene. |
| Mfe_Down_Rv | ATATTCGAGGGGGTTTGGCA | Reverse primer to check knock-out of *MFE* gene. |
| Cex1_Up_Fw | AGCGTCCCTTAAACTCTGGC | Forward primer to check knock-out of *CEX1* gene. |
| Cex1_down_Rv | TGTATCATGCAGGGGCACAA | Reverse primer to check knock-out of *CEX1* gene. |
| ACL_integration_Rv | ACTTCTGGATGTGGTGACCAC | Reverse primer to check insertion of ACL cassette. |
| ACL_integration_Fw | ACTCATGTGGCCGCAAGACTC | Forward primer to check insertion of ACL cassette. |
| ACC_integration_Rv | CTCCTGCTGAGCGATCTCAA | Reverse primer to check insertion of ACC cassette. |
| ACC_integration_Fw | CGTTCTCGACACCCTTGAGA | Forward primer to check insertion of ACC cassette. |
| TS_integration_Rv | GTAGACGTTGGCGTCCTCAA | Reverse primer to check insertion of TS cassette. |
| TS_integration_Fw | AGCCCGCCATTACCATCTTC | Forward primer to check insertion of TS cassette. |
| DGA integration Rv | CGGATCCTTAAGGGCAGGGC | Reverse primer to check insertion of DGA cassette. |
| DGA integration Fw | CCCATGGGCACTCTGGATGACAC | Forward primer to check insertion of DGA cassette. |
| GPD_Fw | ACGAGAACGTGCGATACCTC | Forward primer to check the quality of gDNA in the PCR reaction. |
| GPD_Rv | TTGTCTCCCCAGTTCTTGCC | Reverse primer to check the quality of gDNA in the PCR reaction. |

**Table S2.** Plasmids used to assamble the cassettes for knock-out and overexpress the genetic interventions and built strains.

| **Strain** | **Plasmids used in assembly** | **Genotype/Assembled plasmids** |
| --- | --- | --- |
| *Δmfe* | pSB1A3-GB3 + pCR4Blunt-TOPO-MFE-NotI_Up + pCR4Blunt-TOPO-M-Nat + pCR4Blunt-TOPO-MFE-NotI_Down | pSB1A3-MfeUP-NAT-MfeDOWN |
| *Δcex* | pSB1A3-GB3 + pCRBlunt-CEX1_Up + pCR4Blunt-TOPO-M-hph + pCRBlunt-CEX1_CM_Down | pSB1A3-Cex1UP-HPH-Cex1DOWN |
| *Δmfe*_*acl* | pSB1A3-GB3 + pCR4Blunt-TOPO-MFE-NotI_Up + pCR4Blunt-TOPO-M-Nat+  pCR4Blunt-TOPO- P1 TEF-8UAS + pCRBlunt-ACL + pCR4Blunt-TOPO-TLip2 (E-L) + pCR4Blunt-TOPO-MFE-NotI_Down | pSB1A3-MfeUP-NAT-pTEF8UAS-ACL-tLip2-MfeDOWN |
| *Δmfe*_*acc* | pSB1A3-GB3 + pCR4Blunt-TOPO-MFE-NotI_Up + pCR4Blunt-TOPO-M-Nat+  pCR4Blunt-TOPO- P1 TEF-8UAS + pCRBlunt-ACC + pCR4Blunt-TOPO-TLip2 (E-L) + pCR4Blunt-TOPO-MFE-NotI_Down | pSB1A3-MfeUP-NAT-pTEF8UAS-ACL-tLip2-MfeDOWN |
| *Δmfe*_*ts* | pSB1A3-GB3 + pCR4Blunt-TOPO-MFE-NotI_Up + pCR4Blunt-TOPO-M-Nat+  pCR4Blunt-TOPO- P1 TEF-8UAS + pCRBlunt-TS + pCR4Blunt-TOPO-TLip2 (E-L) + pCR4Blunt-TOPO-MFE-NotI_Down | pSB1A3-MfeUP-NAT-pTEF8UAS-ACL-tLip2-MfeDOWN |
| *Δmfe*_*dga* | pSB1A3-GB3 + pCR4Blunt-TOPO-MFE-NotI_Up + pCR4Blunt-TOPO-M-Nat+  pCR4Blunt-TOPO- P1 TEF-8UAS + pCRBlunt-DGA + pCR4Blunt-TOPO-TLip2 (E-L) + pCR4Blunt-TOPO-MFE-NotI_Down | pSB1A3-MfeUP-NAT-pTEF8UAS-ACL-tLip2-MfeDOWN |
| *Δmfe* _ *Δcex* | pSB1A3-GB3 + pCR4Blunt-TOPO-MFE-NotI_Up + pCR4Blunt-TOPO-M-Nat + pCR4Blunt-TOPO-MFE-NotI_Down | pSB1A3-MfeUP-NAT-MfeDOWN |
|  | pSB1A3-GB3 + pCRBlunt-CEX1_Up + pCR4Blunt-TOPO-M-hph + pCRBlunt-CEX1_CM_Down | pSB1A3-Cex1UP-HPH-Cex1DOWN |
| *Δmfe*_ *Δcex*_*acl* | pSB1A3-GB3 + pCRBlunt-CEX1_Up + pCR4Blunt-TOPO-M-hph + pCRBlunt-CEX1_CM_Down | pSB1A3-Cex1UP-HPH-Cex1DOWN |
|  | pSB1A3-GB3 + pCR4Blunt-TOPO-MFE-NotI_Up + pCR4Blunt-TOPO-M-Nat+  pCR4Blunt-TOPO- P1 TEF-8UAS + pCRBlunt-ACL + pCR4Blunt-TOPO-TLip2 (E-L) + pCR4Blunt-TOPO-MFE-NotI_Down | pSB1A3-MfeUP-NAT-pTEF8UAS-ACL-tLip2-MfeDOWN |
| *Δmfe*_ *Δcex*_*acc* | pSB1A3-GB3 + pCRBlunt-CEX1_Up + pCR4Blunt-TOPO-M-hph + pCRBlunt-CEX1_CM_Down | pSB1A3-Cex1UP-HPH-Cex1DOWN |
|  | pSB1A3-GB3 + pCR4Blunt-TOPO-MFE-NotI_Up + pCR4Blunt-TOPO-M-Nat+  pCR4Blunt-TOPO- P1 TEF-8UAS + pCRBlunt-ACC + pCR4Blunt-TOPO-TLip2 (E-L) + pCR4Blunt-TOPO-MFE-NotI_Down | pSB1A3-MfeUP-NAT-pTEF8UAS-ACC-tLip2-MfeDOWN |
| *Δmfe*_ *Δcex*_*ts* | pSB1A3-GB3 + pCRBlunt-CEX1_Up + pCR4Blunt-TOPO-M-hph + pCRBlunt-CEX1_CM_Down | pSB1A3-Cex1UP-HPH-Cex1DOWN |
|  | pSB1A3-GB3 + pCR4Blunt-TOPO-MFE-NotI_Up + pCR4Blunt-TOPO-M-Nat+  pCR4Blunt-TOPO- P1 TEF-8UAS + pCRBlunt-TS + pCR4Blunt-TOPO-TLip2 (E-L) + pCR4Blunt-TOPO-MFE-NotI_Down | pSB1A3-MfeUP-NAT-pTEF8UAS-TS-tLip2-MfeDOWN |
| *Δmfe*_*Δcex_dga* | pSB1A3-GB3 + pCRBlunt-CEX1_Up + pCR4Blunt-TOPO-M-hph + pCRBlunt-CEX1_CM_Down | pSB1A3-Cex1UP-HPH-Cex1DOWN  pSB1A3-MfeUP-NAT-pTEF8UAS-DGA-tLip2-MfeDOWN |
|  | pSB1A3-GB3 + pCR4Blunt-TOPO-MFE-NotI_Up + pCR4Blunt-TOPO-M-Nat+  pCR4Blunt-TOPO- P1 TEF-8UAS + pCRBlunt-DGA + pCR4Blunt-TOPO-TLip2 (E-L) + pCR4Blunt-TOPO-MFE-NotI_Down |  |
| *Δmfe*_*Δcex_dga_ts* | pSB1A3-GB3 + pCRBlunt-CEX1_Up + pCR4Blunt-TOPO-M-hph + pCR4Blunt-TOPO- P1 TEF-8UAS + pCRBlunt-TS + pCR4Blunt-TOPO-TLip2 (E-L) + pCRBlunt-CEX1_LM_Down | pSB1A3-Cex1UP-HPH-pTEF8UAS-TS-tLip2-Cex1DOWN  pSB1A3-MfeUP-NAT-pTEF8UAS-DGA-tLip2-MfeDOWN |
|  | pSB1A3-GB3 + pCR4Blunt-TOPO-MFE-NotI_Up + pCR4Blunt-TOPO-M-Nat+  pCR4Blunt-TOPO- P1 TEF-8UAS + pCRBlunt-DGA + pCR4Blunt-TOPO-TLip2 (E-L) + pCR4Blunt-TOPO-MFE-NotI_Down |  |

**Table S3.** Fatty acid profile of  *Y. lipolytica* grown at minimal medium with or without supplement (glutamate, methionine, leucine, threonine) at 120h. The fatty acid profile of palm oil was added for direct comparison. MUFAs: Monounsaturated fatty acids, PUFAs: Polyunsaturated fatty acids, VLC-FAs: Very long chain fatty acids.

|  | **Fatty acid profile (%)** | | | | | | | | | | | | | | | | | |
| --- | --- | --- | --- | --- | --- | --- | --- | --- | --- | --- | --- | --- | --- | --- | --- | --- | --- | --- |
| **Supplement** | **C10:0** | **C16:0** | **C16:1** | **C16:2** | **C16:3** | **C18:0** | **C18:1** | **C18:2** | **C18:3** | **C20:0** | **C20:1** | **C22:0** | **C24:0** | **Saturated FAs** | **MUFAs** | **PUFAs** | **VLC-FAs** |  |
| **No supplement** | 0.35 ± 0.04 | 16.23 ± 0.90 | 9.26 ± 0.33 | 0.57 ± 0.02 | 0.70 ± 0.01 | 10.42 ± 0.55 | 37.42 ± 1.27 | 21.14 ± 0.03 | 0.29 ± 0.02 | 0.45 ± 0.01 | - | 0.59 ± 0.02 | 2.59 ± 0.14 | 30.63 ± 1.65 | 46.67 ± 1.61 | 22.70 ± 0.06 | 3.18 ± 0.16 |  |
| **Glutamate** | 0.41 ± 0.05 | 14.45 ± 0.52 | 10.45 ± 0.61 | - | 1.15 ± 0.10 | 11.52 ± 1.00 | 42.92 ± 0.81 | 16.25 ± 1.23 | - | - | - | 0.50 ± 0.06 | 2.34 ± 0.24 | 29.22 ± 1.79 | 53.37 ± 1.31 ** | 17.41 ± 1.17 ** | 2.84 ± 0.30 |  |
| **Methionine** | 0.53 ± 0.02 | 15.71 ± 0.20 | 9.68 ± 0.46 | - | 1.26 ± 0.11 | 10.82 ± 0.33 | 44.14 ± 0.69 | 14.91 ± 0.43 | - | - | - | 0.64 ± 0.08 | 2.32 ± 0.12 | 30.02 ± 0.71 | 53.82 ± 1.12 ** | 16.17 ± 0.54 ** | 2.96 ± 0.19 |  |
| **Leucine** | 0.55 ± 0.12 | 14.86 ± 0.53 | 8.60 ± 0.50 | - | 1.09 ± 0.11 | 13.97 ± 0.89 | 45.64 ± 0.71 | 11.02 ± 0.49 | - | 0.73 ± 0.07 | - | 0.61 ± 0.05 | 2.93 ± 0.08 | 33.65 ± 1.32 | 54.24 ± 0.95 ** | 12.11 ± 0.56 ** | 3.53 ± 0.12 |  |
| **Threonine** | 0.60 ± 0.17 | 15.10 ± 0.39 | 10.14 ± 0.54 | - | 1.14 ± 0.02 | 12.20 ± 0.47 | 44.26 ± 0.50 | 13.31 ± 0.79 | - | - | - | 0.58 ± 0.03 | 2.65 ± 0.16 | 31.14 ± 0.48 | 54.41 ± 0.29 ** | 14.45 ± 0.77 ** | 3.23 ± 0.18 |  |
| **Palm (range)** (Montoya et al., 2013) | - | 32.6–46.0 | 0.0 –0.5 | - | - | 1.7–7.6 | 35.1 – 53.4 | 7.0 –15.0 | 0.2 –0.6 | 0.1–0.5 | 0.1 – 0.2 | – | – | 36.7 –  51.5 | 35.3 – 53.8 | 7.3 – 15.5 | - |  |
| Differences with control (no supplement) were evaluated using a t-test *: *p* ≤ 0.05, **: *p* ≤ 0.01. | | | | | | | | | | | | | | | | | | |

**Table S4.** Lipid content, dry cell weight, lipid weight, biomass, and lipid yield on consumed glycerol of built *Y. lipolytica* strains and extracellular citrate concentrations at C/N140 (g/g) minimal medium (at 120h).

| **Strain** | **Dry cell weight (g/L)** | **Lipid content (%, g/g)** | **Lipid weight (g/L)** | **Y_P/S_**  **(g lipid/g consumed glycerol)** | **Citric acid (g/L)** |  |  |  |
| --- | --- | --- | --- | --- | --- | --- | --- | --- |
|  |  |  |  |  |  |  | |  |
|  |  |  |  |  |  |  | |  |
| WT | 5.47 ± 0.28 | 18.86 ± 0.24 | 1.03 ± 0.07 | 0.026 ± 0.001 | 2.25 ± 0.13 |  | |  |
| *Δcex* | 5.58 ± 0.16 | 18.58 ± 0.41 | 1.04 ± 0.01 | 0.027 ± 0.001 | - |  | |  |
| *Δmfe* | 5.63 ± 0.12 | 19.74 ± 0.33* | 1.11 ± 0.03 | 0.032 ± 0.000 | 2.32 ± 0.1 |  | |  |
| *Δmfe_acl* | 5.85 ± 0.34 | 20.00 ± 0.45 | 1.17 ± 0.08 | 0.030 ± 0.002 | 2.34 ± 0.03 |  | |  |
| *Δmfe_acc* | 5.62 ± 0.14 | 22.39 ± 0.52* | 1.26 ± 0.05 * | 0.031 ± 0.001 | 2.20 ± 0.02 |  | |  |
| *Δmfe_ts* | 5.42 ± 0.18 | 18.06 ± 0.10* | 0.98 ± 0.03 * | 0.026 ± 0.001 | 0.92 ± 0.05** |  | |  |
| *Δmfe_dga* | 6.27 ± 0.27 * | 50.99 ± 1.15** | 3.2 ± 0.14 ** | 0.075 ± 0.004** | 3.33 ± 0.17** |  | |  |
| *Δmfe_Δcex* | 5.23 ± 0.08 | 19.47 ± 0.13* | 1.02 ± 0.01 | 0.029 ± 0.001* | - |  | |  |
| *Δmfe_Δcex_acl* | 5.42 ± 0.16 | 22.38 ± 0.10** | 1.21 ± 0.04 ** | 0.032 ± 0.001 | 1.08 ± 0.09 ** |  | |  |
| *Δmfe_Δcex_acc* | 5.49 ± 0.13 | 22.17 ± 0.38** | 1.22 ± 0.02 ** | 0.033 ± 0.001* | 1.05 ± 0.04 ** |  | |  |
| *Δmfe_Δcex_ts* | 5.48 ± 0.23 | 22.45 ± 0.17** | 1.23 ± 0.05 ** | 0.033 ± 0.002 | 0.66 ± 0.03 ** |  | |  |
| *Δmfe_Δcex_dga* | 6.34 ± 0.14 * | 53.53 ± 0.53** | 3.40 ± 0.10 ** | 0.089 ± 0.004** | - |  | |  |
| *Δmfe_Δcex_dga_ts* | 6.17 ± 0.10 * | 55.87 ± 0.62** | 3.45 ± 0.04 ** | 0.085 ± 0.001** | - |  | |  |
| Differences with control (background strains compared to WT and other transformants compared to the background strain) were evaluated using a t-test *: *p* ≤ 0.05, **: *p* ≤ 0.01. | | | | | | |  | |

**Table S5.** Measured erythritol and arabitol concentration of *Y. lipolytica* strains and at C/N140 (g/g) minimal medium (at 120h).

| **Strain** | **Mannitol (g/L)** | **Erythritol (g/L)** | **Arabitol (g/L)** |
| --- | --- | --- | --- |
| WT | 18.75 ± 1.47 | 1.00 ± 0.09 | 0 ± 0 |
| *Δcex* | 16.87 ± 0.87 | 1.44 ± 0.09 ** | - |
| *Δmfe* | 15.35 ± 1.13 | 0.92 ± 0.03 | - |
| *Δmfe_acl* | 15.53 ± 0.25 | - | - |
| *Δmfe_acc* | 16.26 ± 0.10 | 0.85 ± 0.01 | - |
| *Δmfe_ts* | 18.22 ± 0.40 | 1.23 ± 0.04 ** | - |
| *Δmfe_dga* | 8.69 ± 0.14 ** | 0.14 ± 0.00 ** | 3.52 ± 0.18 |
| *Δmfe_Δcex* | 15.21 ± 0.10 * | 1.43 ± 0.03 ** | - |
| *Δmfe_Δcex_acl* | 11.42 ± 0.99 ** | 3.20 ± 0.08 ** | - |
| *Δmfe_Δcex_acc* | 10.50 ± 0.73 ** | 3.31 ± 0.05 ** | - |
| *Δmfe_Δcex_ts* | 10.35 ± 0.35 ** | 3.28 ± 0.06 ** | - |
| *Δmfe_Δcex_dga* | 8.34 ± 0.04 ** | 0.96 ± 0.03 ** | 2.19 ± 0.02 |
| *Δmfe_Δcex_dga_ts* | 8.29 ± 0.54 ** | 0.25 ± 0.03 ** | 1.60 ± 0.11 |
| Differences with control (background strains compared to WT and other transformants compared to the background strain) were evaluated using a t-test *: p ≤ 0.05, **: p ≤ 0.01. | | | |

**Table S6.** Fatty acid profile of transformants and WT grown at C/N140 at 120 h. The fatty acid profile of palm oil was added for direct comparison. MUFAs: Monounsaturated fatty acids, PUFAs: Polyunsaturated fatty acids. Differences with control (WT) were evaluated using a t-test *: *p* ≤ 0.05, **: *p* ≤ 0.01.

|  | **Fatty Acid Profile (%)** | | | | | | | | | | | | | | | | | | |
| --- | --- | --- | --- | --- | --- | --- | --- | --- | --- | --- | --- | --- | --- | --- | --- | --- | --- | --- | --- |
| **Strain** | **C10:0** | **C14:0** | **C16:0** | **C16:1** | **C16:2** | **C16:3** | **C18:0** | **C18:1** | **C18:2** | **C18:3** | **C20:0** | **C20:1** | **C22:0** | **C22:1** | **C24:0** | **Saturated FA** | **MUFAs** | **PUFAs** | **VLCFAs** |
| **WT** | 0.35 ± 0.01 | 0.27 ± 0.02 | 16.63 ± 0.31 | 9.31 ± 0.32 | 0.55 ± 0.04 | 0.75 ± 0.02 | 10.86 ± 0.29 | 36.34 ± 0.22 | 20.75 ± 0.37 | 0.24 ± 0.01 | 0.42 ± 0.01 | 0.85 ± 0.15 | 0.56 ± 0.01 | - | 2.13 ± 0.15 | 31.21 ± 0.53 | 46.50 ± 0.32 | 22.29 ± 0.34 | 2.68 ± 0.14 |
| ***Δcex*** | 0.42 ± 0.05 | 0.34 ± 0.05 | 16.49 ± 1.17 | 10.34 ± 0.33 | 0.45 ± 0.01 | 0.69 ± 0.02 | 8.39 ± 0.48 | 39.21 ± 0.20 | 21.12 ± 0.20 | - | 0.53 ± 0.01 | 0.65 ± 0.06 | 0.40 ± 0.01 | - | 2.29 ± 0.21 | 28.86 ± 0.62 * | 50.20 ± 0.49 ** | 22.34 ± 0.62 | 2.69 ± 0.20 |
| ***Δmfe*** | 0.22 ± 0.06 | 0.44 ± 0.09 | 16.76 ± 0.73 | 9.11 ± 0.33 | 0.35 ± 0.01 | 0.88 ± 0.05 | 11.36 ± 0.51 | 37.68 ± 0.69 | 20.03 ± 0.45 | 0.27 ± 0.02 | 0.69 ± 0.03 | 0.49 ± 0.03 | 0.61 ± 0.03 | - | 1.12 ± 0.04 | 31.20 ± 1.42 | 47.28 ± 1.03 | 21.52 ± 0.39 | 1.73 ± 0.06 ** |
| ***Δmfe_acl*** | 0.85 ± 0.10 | 0.24 ± 0.01 | 18.11 ± 0.47 | 11.41 ± 0.19 | 0.26 ± 0.03 | 0.79 ± 0.02 | 9.03 ± 0.25 | 37.38 ± 0.06 | 20.17 ± 0.58 | - | - | - | - | - | 1.77 ± 0.06 | 29.99 ± 0.62 | 48.79 ± 0.14 ** | 21.23 ± 0.54 | 1.77 ± 0.06 ** |
| ***Δmfe_acc*** | 0.30 ± 0.02 | 0.18 ± 0.01 | 14.81 ± 0.35 | 12.58 ± 0.39 | 0.25 ± 0.01 | 0.68 ± 0.03 | 6.46 ± 0.34 | 41.89 ± 0.28 | 21.67 ± 0.20 | - | - | - | - | - | 1.21 ± 0.04 | 22.94 ± 0.57 ** | 54.47 ± 0.45 ** | 22.59 ± 0.17 | 1.21 ± 0.04 ** |
| ***Δmfe_ts*** | 0.62 ± 0.03 | 0.24 ± 0.01 | 14.67 ± 0.54 | 12.93 ± 0.16 | 0.12 ± 0.01 | 0.69 ± 0.01 | 6.60 ± 0.24 | 47.83 ± 0.69 | 15± 0.43 | - | - | - | - | - | 1.30 ± 0.16 | 23.43 ± 0.30 ** | 60.76 ± 0.73 ** | 15.81 ± 0.43 ** | 1.30 ± 0.16 ** |
| ***Δmfe_dga*** | - | 0.27 ± 0.01 | 14.74 ± 0.14 | 6.64 ± 0.05 | 0.14 ± 0.00 | 0.53 ± 0.01 | 15.21 ± 0.10 | 50.16 ± 0.16 | 8.91 ± 0.11 | 0.14 ± 0.00 | 0.81 ± 0.03 | 0.53 ± 0.04 | 0.71 ± 0.01 | 0.27 ± 0.02 | 0.96 ± 0.06 | 32.71 ± 0.25 * | 57.60 ± 0.12 ** | 9.72± 0.11 ** | 1.94 ± 0.05 ** |
| ***Δmfe_Δcex*** | 0.44 ± 0.04 | 0.52 ± 0.04 | 16.95 ± 0.33 | 10.57 ± 0.15 | 0.28 ± 0.01 | 0.86 ± 0.05 | 10.86 ± 0.33 | 41.40 ± 0.68 | 14.85 ± 0.33 | - | 0.65 ± 0.03 | 0.59 ± 0.04 | 0.58 ± 0.01 | - | 1.46 ± 0.12 | 31.45 ± 0.75 | 52.56 ± 0.86 ** | 15.99 ± 0.32 ** | 2.04 ± 0.13 ** |
| ***Δmfe_Δcex_acl*** | 0.49 ± 0.13 | 0.32 ± 0.01 | 15.11 ± 0.71 | 11.79 ± 0.51 | 0.66 ± 0.05 | 0.73 ± 0.03 | 5.71 ± 0.26 | 41.97 ± 0.84 | 19.51 ± 0.36 | 0.58 ± 0.00 | - | 0.61 ± 0.02 | 0.31 ± 0.01 | - | 2.20 ± 0.13 | 24.15 ± 0.35 ** | 54.37 ± 0.51 ** | 21.48 ± 0.40 | 2.51 ± 0.13 |
| ***Δmfe_Δcex_acc*** | 0.41 ± 0.00 | 0.36 ± 0.02 | 15.30 ± 0.29 | 10.94 ± 0.30 | 0.28 ± 0.01 | 0.69 ± 0.05 | 6.24 ± 0.58 | 43.54 ± 0.30 | 18.94 ± 0.12 | 0.56 ± 0.03 | - | 0.66 ± 0.08 | 0.30 ± 0.00 | - | 1.93 ± 0.09 | 24.53 ± 0.49 ** | 55.13 ± 0.52 ** | 20.47 ± 0.17 ** | 2.23 ± 0.10 * |
| ***Δmfe_Δcex_ts*** | 0.42 ± 0.02 | 0.36 ± 0.02 | 15.49 ± 0.05 | 10.90 ± 0.23 | 0.61 ± 0.02 | 0.71 ± 0.03 | 6.92 ± 0.16 | 42.13 ± 0.74 | 19.18 ± 0.52 | 0.53 ± 0.09 | - | 0.56 ± 0.01 | 0.30 ± 0.00 | - | 2.14 ± 0.13 | 25.62 ± 0.33 ** | 53.59 ± 0.54 ** | 21.02 ± 0.44 * | 2.44 ± 0.13 |
| ***Δmfe_Δcex_dga*** | - | 0.20 ± 0.01 | 16.39 ± 0.58 | 6.65 ± 0.29 | - | 0.33 ± 0.03 | 14.14 ± 0.53 | 51.65 ± 0.30 | 5.40 ± 0.30 | - | 0.85 ± 0.02 | 1.15 ± 0.04 | 0.76 ± 0.01 | 0.52 ± 0.06 | 1.91 ± 0.13 | 34.26 ± 0.23 ** | 59.97 ± 0.57 ** | 5.89 ± 0.31 ** | 3.19 ± 0.08 * |
| ***Δmfe_Δcex_dga_ts*** | - | 0.22 ± 0.01 | 15.15 ± 0.33 | 6.48 ± 0.23 | 0.17 ± 0.00 | 0.43 ± 0.01 | 14.50 ± 0.23 | 49.80 ± 0.44 | 8.71 ± 0.17 | 0.11 ± 0.01 | 1.05 ± 0.03 | 0.57 ± 0.02 | 0.80 ± 0.03 | 0.32 ± 0.01 | 1.79 ± 0.12 | 33.50 ± 0.58 * | 57.16 ± 0.59 ** | 9.42 ± 0.19 ** | 2.90 ± 0.14 |
| **Palm (range)**  (Montoya et al., 2013) | - |  | 32.6–46.0 | 0.0 –0.5 | - | - | 1.7–7.6 | 35.1 – 53.4 | 7.0 –15.0 | 0.2 –0.6 | 0.1–0.5 | 0.1 – 0.2 | – |  | – | 36.7 – 51.5 | 35.3 – 53.8 | 7.3 – 15.5 |  |
| Differences with control (WT) were evaluated using a t-test *: p ≤ 0.05, **: p ≤ 0.01. | | | | | | | | | | | | | | | | | | | |

**Table S7.** Model coefficients, and statistics of quadratic regression model.

| **Dependent variable: Lipid content % (w/w)** | | | |
| --- | --- | --- | --- |
| **Source** | **Estimate** | **Std. Error** | **p-value** |
| Intercept | 37.78 | 0.3838 | < e-16 |
| *DGA* | 16.17 | 0.1637 | < e-16 |
| *CEX1* | 2.41 | 0.4330 | < e-06 |
| *ACC* | 1.34 | 0.1637 | < e-08 |
| *TS:CEX1* | 1.17 | 0.1637 | < e-07 |
| *ACL* | 0.79 | 0.1637 | < e-05 |
| *DGA:CEX1* | 0.70 | 0.1637 | < e-04 |
| *ACL:CEX1* | 0.66 | 0.1637 | < e-04 |
| *MFE* | 0.44 | 0.1637 | 0.01 |
| *TS* | 0.16 | 0.1637 | 0.32 |
| *ACC:CEX1* | 0.01 | 0.1637 | 0.93 |
| *MFE:CEX1* | 0.00 | 0.1637 | 0.99 |
| *TS:DGA* | -0.16 | 0.1637 | 0.34 |
| R^2^: 0.9989, Adj R^2^:0.9984, p-value: < 2.2e-16 | | | |
